# Antitumoral Effect of Plocabulin in High Grade Serous Ovarian Carcinoma Cell Line Models

**DOI:** 10.3389/fonc.2022.862321

**Published:** 2022-03-17

**Authors:** Victoria Heredia-Soto, Javier Escudero, María Miguel, Patricia Ruiz, Alejandro Gallego, Alberto Berjón, Alicia Hernández, Marta Martínez-Díez, Shuyu Zheng, Jing Tang, David Hardisson, Jaime Feliu, Andrés Redondo, Marta Mendiola

**Affiliations:** ^1^ Translational Oncology Research Laboratory, Hospital La Paz Institute for Health Research (IdiPAZ), Madrid, Spain; ^2^ Center for Biomedical Research in the Cancer Network (Centro de Investigación Biomédica en Red de Cáncer, CIBERONC), Instituto de Salud Carlos III, Madrid, Spain; ^3^ Department of Medical Oncology, Hospital Universitario La Paz, Madrid, Spain; ^4^ Department of Pathology, Hospital Universitario La Paz, Madrid, Spain; ^5^ Molecular Pathology and Therapeutic Targets Group, Hospital La Paz Institute for Health Research (IdiPAZ), Madrid, Spain; ^6^ Department of Obstetrics and Gynecology, Hospital Universitario La Paz, Madrid, Spain; ^7^ Faculty of Medicine, Universidad Autónoma de Madrid, Madrid, Spain; ^8^ Cell Biology Department, Research and Development, Oncology Business Unit, Pharmamar, Madrid, Spain; ^9^ Research Program in Systems Oncology, Faculty of Medicine, University of Helsinki, Helsinki, Finland; ^10^ Cátedra UAM-ANGEM, Faculty of Medicine, Universidad Autónoma de Madrid, Madrid, Spain

**Keywords:** plocabulin (PM060184), microtubule inhibitor, high-grade serous ovarian cancer (HGSOC), 3D cell culture, drug testing

## Abstract

Ovarian cancer (OC) is a life-threatening tumor and the deadliest among gynecological cancers in developed countries. First line treatment with a carboplatin/paclitaxel regime is initially effective in the majority of patients, but most advanced OC will recur and develop drug resistance. Therefore, the identification of alternative therapies is needed. In this study, we employed a panel of high-grade serous ovarian cancer (HGSOC) cell lines, in monolayer and three-dimensional cell cultures. We evaluated the effects of a novel tubulin-binding agent, plocabulin, on proliferation, cell cycle, migration and invasion. We have also tested combinations of plocabulin with several drugs currently used in OC in clinical practice. Our results show a potent antitumor activity of plocabulin, inhibiting proliferation, disrupting microtubule network, and decreasing their migration and invasion capabilities. We did not observe any synergistic combination of plocabulin with cisplatin, doxorubicin, gemcitabine or trabectedin. In conclusion, plocabulin has a potent antitumoral effect in HGSOC cell lines that warrants further clinical investigation.

## Introduction

Ovarian cancer (OC) is the leading cause of death for patients with gynecological malignancies. It is an indolent disease, frequently diagnosed at advanced stages due to the lack of specific symptoms. For decades, treatment of OC has consisted of surgery and systemic adjuvant or neoadjuvant chemotherapy with a carboplatin/paclitaxel regimen. However, despite achieving initial complete remission, about 80% of patients with advanced disease will relapse and finally progress to a platinum-resistant OC (1).

Platinum response is one of the major prognostic factors in OC. The classical classification of recurrence in platinum-sensitive or platinum-resistant/refractory disease has been based on the cut-off of 6 months after completing chemotherapy, and no validated biomarkers, other than histological subtype, are known to predict likelihood of primary platinum-resistant or platinum-refractory disease (2). Few single agents have shown discrete activity in platinum-resistant OC, such as weekly paclitaxel, pegylated liposomal doxorubicin (PLD), gemcitabine, topotecan, cyclophosphamide or etoposide. The response to these single agents is usually less than 20%, with a median progression-free survival (PFS) less of 6 months and a median overall survival (OS) around 12 months (3).

In recent years some relevant progress has occurred in the treatment of high grade serous ovarian carcinoma (HGSOC), the most prevalent subtype of OC, with the introduction of poly-adenosine diphosphate ribose polymerase inhibitors (PARPi). These targeted therapies are now being administered as maintenance therapy after chemotherapy, achieving a relevant improvement in PFS, not only after first line chemotherapy (4–6), but also after platinum-sensitive relapse (7–9). However, the efficacy of current treatments remains limited, especially in platinum-resistant/refractory disease, and there is still a medical unmet need for testing and developing novel therapies for OC patients after progression to the current options.

Plocabulin (PM060184/PM184, PharmaMar) is a compound of marine origin derived from the Madagascan sponge *Lithoplocamia lithistoides*. Plocabulin belongs to a family of tubulin-binding agents that inhibits tubulin polymerization by binding to the dimer’s end, with one of the highest known affinities among tubulin-binding agents. This mechanism alters the dynamic instability of microtubules and affects cells both in interphase and mitosis, inhibiting cell growth and migration (10–12). Recent studies have also demonstrated an antiangiogenic effect of plocabulin, which causes a reduction in vascular volume and induction of necrosis both *in vitro* and *in vivo* (13).

Phase I studies have demonstrated promising antitumor effects of plocabulin in patients with advanced tumors and, currently, it is being further assessed in clinical trials in advanced colorectal cancer, breast cancer and other solid tumors (11, 14).

In the current study, we explore for the first time the *in vitro* efficacy of plocabulin in a panel of 12 HGSOC cell lines with distinct sensitivities to cisplatin (CDDP), alone and in combination with other drugs currently applied in clinical practice. Studies have been done both in monolayer culture (2D) and three dimensional (3D) spheroids, a promising preclinical model for testing antitumor drugs.

## Material and Methods

### Cell Lines and Culture Conditions

PEA1, PEA2, PEO1, PEO4, PEO6, PEO14, PEO23, PEO16, OVCAR-3 and 59M cell lines were obtained from the European Collection of Authenticated Cell Cultures (ECACC), and cultured following the guidelines of the repository. OV866(2) and TOV3041G were obtained from Centre Hospitalier de L´Université de Montréal (CHUM), and kindly provided by Dr. Mes-Masson.

Some of these lines were established from the same patient during the course of disease and had received different treatment schemes prior to their establishment: PEA1/PEA2, PEO1/PEO4/PEO6 and PEO14/PEO23 (15). PEO1 and PEO16 harbor reported deleterious mutations in *BRCA2* (16). As previously reported by our group, four resistance groups were established according to their CDDP IC_50_ values (17). **Table 1** shows the treatment administered to the patient prior to the establishment of the cell line and the CDDP resistance group assigned.

**Table 1:** Cell line characteristics. Previous treatments received by the patients and CDDP sensitivity group based on our previous report (17).

Cell line	Previous treatments	CDDP sensitivity
**PEA1**	NO	VR
**PEA2**	CDDP, PREDNIMUSTIN	VR
**PEO1**	CDDP, 5-FU, CHLORAMBUCIL	PR
**PEO4**	CDDP, 5-FU, CHLORAMBUCIL	VR
**PEO6**	CDDP, 5-FU, CHLORAMBUCIL	R
**PEO14**	NO	S
**PEO23**	CDDP, CHLORAMBUCIL	R
**PEO16**	RADIOTHERAPY	PR
**OVCAR-3**	CYCLOPHOSPHAMIDE, ADRIAMYCIN, CDDP	PR
**OV866(2)**	CARBOPLATIN, TAXOL	VR
**TOV3041 G**	CDDP, CARBOPLATIN, TAXOL	PR
**59M**	NO	R

CDDP, cisplatin, 5-FU, 5-Fluorouracil; S, sensitive; PS, partially resistant; R, resistant; VR, very resistant.

Cells were maintained in the following culture media: PEA1, PEA2, PEO14, PEO23, PEO16 and OVCAR-3: Roswell Park Memorial Institute (RPMI); PEO1, PEO4, PEO6 and 59M: Dulbecco’s Modified Eagle Medium (DMEM), both supplemented with 10% Fetal Bovine Serum (FBS) and 100 U/ml penicillin–streptomycin. OV866(2) and TOV3041G were grown in a combination of 199 and MCDB105 (1:1) media with 5% FBS and 50 μg/ml gentamicin (Merck, MA, USA). All cells were incubated at 37°C in a 5% CO_2_ incubator.

All cell lines were tested periodically for mycoplasma infection and authenticated by genetic profiling using polymorphic short tandem repeat loci with the Geneprint 10 kit (Promega, WI, USA).

### Drug Treatment Assays

For monolayer culture experiments, cells were seeded in flat bottom 96-well plates (Corning, NY, USA) 24 hours before drug exposure (cell density was previously calculated for each cell line to avoid confluence at the final time point). Then, cells were exposed to different drug concentrations for 72 hours. After this time, cellular confluence was measured with sulforhodamine B (SRB) colorimetric assay.

In the case of 3D culture, spheroids were cultured using ultra-low attachment (ULA) plates (Corning) as previously described (17, 18). Cell density was previously calculated so that the spheroids had a diameter of 300–400 μm at day 4, optimal to mimic the diffusion state in the tumor, which is about 100 μm in depth for nutrients and oxygen, avoiding excessive necrotic areas. In these experiments, after 4 days of culture, spheroids were exposed to plocabulin for 72 hours. Cell viability was then measured using CellTiter-Glo (CTG) Luminescent assay (Promega).

Colorimetry (SRB) and luminescence (CTG) were measured using a Synergy 4 microplate reader (BioTek, VT, USA), and in both cases half maximal inhibitory concentration (IC_50_) values were calculated using linear regression with GraphPad Prism 7 software (GraphPad Software, CA, USA).

Possible synergisms were assayed, in 2D conditions, between plocabulin and other chemotherapeutic agents currently administered to patients with HGSOC in routine clinical practice: CDDP, doxorubicin, gemcitabine and trabectedin. For these assays, we selected 7 cell lines with different sensitivities to CDDP, with or without a previous treatment. These cells were: PEA1, PEA2, PEO4, PEO14, OV866(2) and TOV3041G, which covered all CDDP possible scenarios.

**Figure 1:**
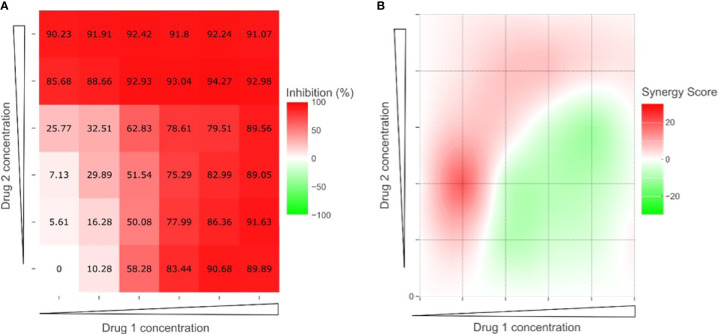
SynergyFinder Plus analysis for an example of two-drug combinations. **(A)** Dose-response matrix calculated as percentage of growth inhibition. **(B)** Synergy distribution map; red indicates synergy (synergy score > 0) and green indicates antagonism (synergy score < 0).

The experimental design was based on the evaluation of two drugs on a 6x6 matrix (**Figure 1A**). One of the drugs is dosed by increasing concentrations by row, and the other one by column. With this design, the bottom left well corresponds to the control without drugs, and the top right well corresponds to the maximum combined concentration of both drugs. First row and the first column correspond to single drugs, and the remaining wells contain increasing drug combinations, each well with a different dose.

We used the SynergyFinder Plus web tool (https://synergyfinder.org) to explore the synergistic effects of plocabulin with the other drugs (19). This tool applies four different algorithms: ZIP (zero interaction potency), HSA (highest single agent), Bliss (Bliss independence) and Loewe (Loewe additivity) (20, 21). To increase the robustness of the analysis we decided that only if the four algorithms showed global positive results (synergy score > 10), we could confirm the existence of synergy between the two drugs (**Figure 1**) (22).

CDDP, doxorubicin and gemcitabine were provided by the pharmacy of the Hospital La Paz. Plocabulin and trabectedin were kindly provided by PharmaMar (Madrid, Spain).

### Invasion and Migration Assays

Invasion and migration capacity was evaluated in 2D and 3D conditions.

For monolayer culture experiments, cells were treated with plocabulin for 72 hours and then transferred into transwell inserts in low serum conditions (1% in the top chamber and basal FBS conditions in the bottom chamber to induce cell mobility). 8 μm pore transwell inserts were employed for migration assays, and pre-coated inserts with Matrigel^®^ were used for invasion experiments (Corning). Twenty-four hours later, cells were removed from the top chamber using a cotton swab and the inserts were fixed by the Diff-Quick method (QCA, Tarragona, Spain). Pictures of the inserts were taken and cells were counted manually with ImageJ (NIH, MD, USA) using a representative area of each well.

In 3D conditions cells were plated in ULA plates and spheroids let to grow for four days. At day 4, plocabulin was added at the correspondent IC_50_ dose for 72 hours. After this time, for invasion assays, drugs were removed and Matrigel^®^ (1:3 with culture media) was added to the spheroids. Pictures were taken every day. For migration assays, spheroids were transferred onto a Matrigel^®^ layer, and pictures were taken as done for invasion assays.

### Microtubule Network and Mitotic Spindle Staining by Immunofluorescence

For the study of microtubules, immunofluorescence staining was performed on OV866(2) cell line for α- and γ-tubulin. Briefly, cells were seeded on glass coverslips and 24 hours later were exposed to different concentrations of plocabulin (control, 0.1 nM, and 1 nM) for 48 hours. After this time, two different IF approaches were done:

- Detection of alterations in microtubules and mitotic spindle: Cells were fixed with methanol for 10 minutes at -20°C and incubated with a blocking solution (5% bovine serum albumin in PBS) for 30 minutes and incubated with the corresponding primary and secondary antibodies, as previously described (11).

Pictures were taken with a Leica DM IRM fluorescence microscope equipped with a 100X oil immersion objective and a DFC 340 FX digital camera (Leica, Wetzlar, Germany). Micronuclei were scored in a minimum of 5 fields for each treatment condition.

Antibody and Hoechst details are listed on **Supplementary Table 1**.

### Cell Cycle Assays

For cell cycle studies, OV866(2) cells were exposed to 0.1 or 1 nM concentrations of plocabulin for 24h. After this time, cells were fixed with 70% ice-cold ethanol for 15 minutes at 4°C and then stained with a propidium iodide solution (Merck) for 30 minutes, in the dark, at room temperature. After washing with phosphate buffered saline, cells were analyzed for cell cycle on a Celigo S plate cytometer (Nexcelom, MA, USA).

### Statistical Analysis

Statistical analysis of all experiments was carried out by means of the Student’s T-test using Microsoft Excel (Microsoft, WA, USA). Statistical significance is reported when p-value ≤ 0.05.

All experiments were performed at least in duplicate.

## Results

### IC_50_ Determination

**Table 2** shows the IC_50_ values determined for each cell line and drug in 2D and 3D conditions.

**Table 2:** IC_50_ values for plocabulin (PM060184) in 2D and 3D conditions.

Cell Line	PM060184 (nM)				Ratio PM 3D/2D
	IC_50_ 2D	Std. Dev.	IC_50_ 3D	Std. Dev.	
**PEA1**	0.07	0.04	> 10	N/D	N/D
**PEA2**	0.23	0.04	> 10	N/D	N/D
**PEO1**	0.03	0.01	> 10	N/D	N/D
**PEO4**	0.05	0.02	0.16	0.05	2.95
**PEO6**	0.37	0.05	0.24	0.13	0.65
**PEO14**	> 10	N/D	> 10	N/D	N/D
**PEO23**	0.35	0.08	> 10	N/D	N/D
**PEO16**	0.30	0.36	0.05	0.02	0.17
**OVCAR-3**	0.03	0.01	> 10	N/D	N/D
**OV866(2)**	0.08	0.05	> 10	N/D	N/D
**TOV3041 G**	0.07	0.02	> 10	N/D	N/D
**59M**	1.15	0.09	> 10	N/D	N/D

Std. Dev., Standard deviation; N/D, not determined. N/D values were not calculated since an IC_50_ value was not reached, therefore standard deviations or ratios cannot be performed. Data are represented as mean and standard deviation. 3D/2D ratio has been calculated for cell lines when both IC_50_ values were available.

When cultured in monolayer, plocabulin was effective in 11/12 cell lines, at doses in low nanomolar/picomolar range (< 1.2 nM), as we can see in the IC_50_ values obtained. Only PEO14 was resistant to a concentration of 10 nM. Besides PEO14, 59M showed the highest IC_50_ value for plocabulin; they are both chemo naïve cell lines but sensitive (PEO14) or resistant (59M) to CDDP, according to our sensitivity stratification. We could not correlate plocabulin response with previous treatments or *BRCA* status, but it showed antitumoral activity in all CDDP sensitivity groups.

However, in 3D spheroids only PEO4, PEO6 and PEO16 showed a response to plocabulin in a low nanomolar range. Of these cells, as we can see in the ratio PM 3D/2D column, PEO4 was almost 3 times more resistant to plocabulin in 3D conditions, whilst PEO6 and PEO16 were sensitized to plocabulin in these culture conditions. All the other cell lines had IC_50_ values over 10 nM, which is 100–1000 times more resistance than in monolayer culture (except for 59M, where the increase was only 10 times).

### Invasion and Migration Assays

Cell mobility assays were performed in 7 cell lines, chosen by their different sensitivity to CDDP: PEA1, PEA2, PEO1, PEO4, PEO14, PEO16 and OV866(2).

**Figure 2:**
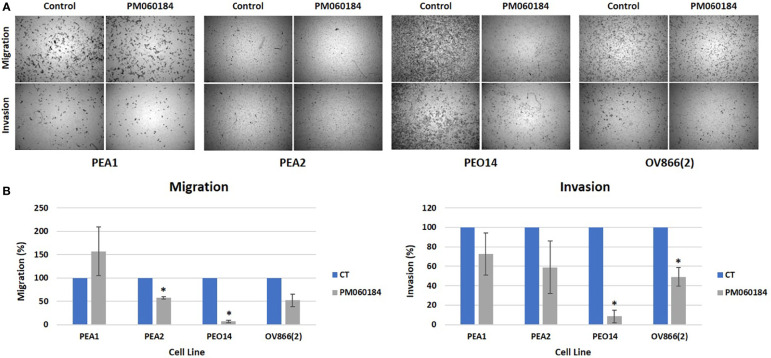
Plocabulin effect on 2D migration and invasion. **(A)** Transwell migration and invasion images of a representative experiment of PEA1, PEA2, PEO14 and OV866(2) cells. **(B)** Bar plots represent the quantification of the data calculated with the mean values of at least two experiments. CT: untreated control; *: p value < 0.05.

We observed a reduction of both transwell migration (3 cell lines) and invasion (4 cell lines) when treating cells with plocabulin (**Figure 2**). The highest inhibition of migration was achieved in PEO14 (93.3% inhibition, p value = 0.02), followed by OV866(2) (48.1% inhibition, p value = 0.07) and PEA2 (42.6% inhibition, p value = 0.04), with the exception of PEA1, where we saw a non significant increase of migration. Regarding invasion through a Matrigel^®^ layer, again, we saw the highest inhibition in PEO14 (91.6% inhibition, p value = 0.04), followed by OV866(2) (50.9% inhibition, p value = 0.04), PEA2 (41.1% inhibition, p value = 0.37) and PEA1 (27.3% inhibition, p value = 0.43). The latter, although not significant, show a similar trend towards inhibition of migration and invasion (**Figure 2B**). PEO1, PEO4 and PEO16 were not evaluable, since they did not invade or migrate through the transwell inserts.

**Figure 3:**
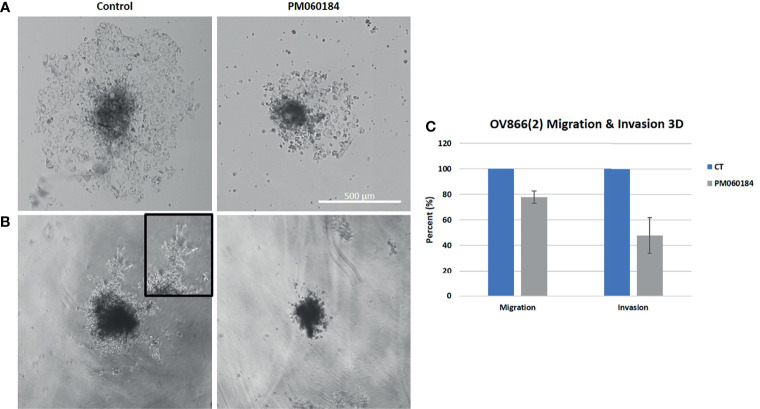
Plocabulin effect on OV866(2) spheroids migration **(A)** and invasion **(B)**. Pictures are of a representative experiment. **(C)** Bar plots represent the quantification of the data calculated with the mean values of at least two experiments. CT: untreated control.

In 3D experiments, only cell lines that make either compact aggregates (PEO1, PEO4 and PEO14) or tight spheroids (OV866(2) and PEO16) were assayed, since loose aggregates (PEA1 and PEA2) cannot be transferred to Matrigel^®^ without disintegration. Of all these selected cell lines, only OV866(2) migrated (**Figure 3A**) and invaded (**Figure 3B**) in basal conditions when transferred to Matrigel^®^, and this behavior was partially inhibited when cells were treated with plocabulin. Plocabulin reduces spheroid volume and spread both in invasion and migration experiments. Migratory spread was reduced by 22.7%, while invasion was reduced by 56.6%, although these results did not reach statistical significance (p values = 0.14 and 0.17, respectively) (**Figure 3C**).

### Immunofluorescence

The effect of plocabulin treatment on the microtubule network of OV866(2) cells was analyzed by immunofluorescence staining of α- and γ-tubulin.

**Figure 4:**
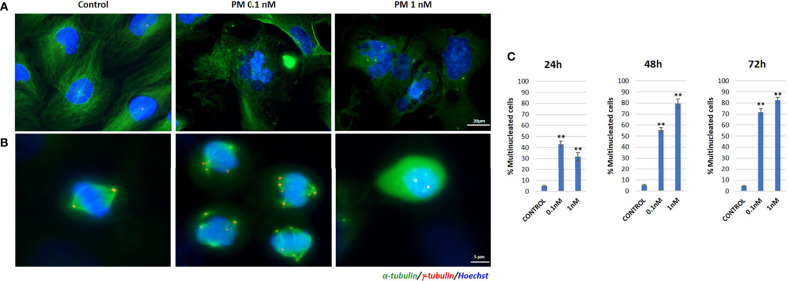
Immunofluorescence staining of α- and γ-tubulin in plocabulin treated OV866(2) cells. **(A)** Effects of 48h treatment with plocabulin on microtubule network and appearance of multinucleated cells. **(B)** Aberrant mitotic spindle polarization and chromosome missegregation after treatment with plocabulin. **(C)** Bar plots represent percentage of multinucleated cells after treatment with plocabulin (0.1 and 1 nM) at 24, 48 and 72h. PM: PM060184; **: p value < 0.01.

**Figure 5:**
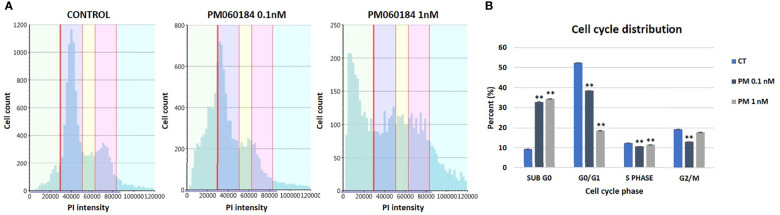
Cell cycle experiments in OV866(2) cells. **(A)** Cell cycle diagrams obtained by Celigo S plate cytometer of OV866(2) cells treated for 24h with plocabulin at 0.1nM and 1nM, and the untreated control. **(B)** Bar plots represent the percentage of cells at each phase of the cell cycle. CT: untreated control; PM: PM060184; PI: propidium iodide; **: p value < 0.01.

Plocabulin treatment induced microtubule depolymerization in a concentration-dependent manner. At IC_50_ value doses (0.1 nM), microtubule distribution was slightly disorganized, and this effect was accentuated at 1 nM (**Figure 4A**). Treatment with plocabulin also caused the appearance of aberrant mitoses, chromosome missegregation and multinucleated cells in a concentration-dependent manner. In untreated cells, mitoses showed a bipolar spindle and chromosome alignment at the metaphase plate. 24h treatment with plocabulin produced an increase of multinucleated cells; at 0.1 nM, 43.0% presented micronuclei, versus 5.2% in control cells (p value < 0.001) while 31.8% (p value < 0.001) were multinucleated at 1 nM of plocabulin (**Figures 4A, C**). The percentage of multinucleated cells increased in a statistically significant manner (p value < 0.001) with time and drug concentration (**Figure 4C**) data that suggest an apoptotic death of these cells. Cell cycle disruption and an increase of apoptosis were also seen in cell cycle experiments, in conjunction with a decrease of G_0_/G_1_ phase (**Figure 5**).

### Synergisms

**Figure 6:**
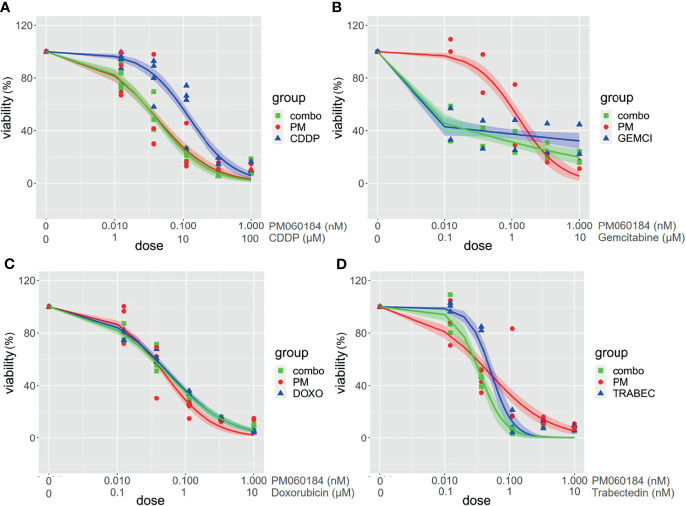
Drug combination assays in OV866(2) cell line. Graphs represent viability curves for individual drugs (PM060184: red; cisplatin (CDDP), doxorubicin, gemcitabine or trabectedin: blue, and for combinations: green). Range concentration of each drug are described in the X-axis. PM060184 was combined with **(A)** CDDP, **(B)** Gemcitabine, **(C)** Doxorubicin, and **(D)** Trabectedin.

We did not find any clear synergism in the four combinations tested in any of the cell lines (PEA1, PEA2, PEO4, PEO14, OV866(2) and TOV3041G). Although some combinations showed punctual synergy at individual dose combinations, overall, none of the experiments showed a positive synergy score for all four algorithms analyzed in SynergyFinder Plus (**Supplementary Table 2**). Nor did we observe any additive effect reducing the effective dose of the other agent tested. **Figure 6** shows an example of these drug response curves in OV866(2) cell line.

## Discussion

Plocabulin is a novel tubulin-binding agent of marine origin that has been proven to potently disrupt cellular microtubules and mitosis and thus inhibit the proliferation of tumor cell lines (12). In the present study we have investigated the antiproliferative effect of plocabulin in a panel of HGSOC cell lines, including CDDP resistant scenario, and our results demonstrate that plocabulin has a dose-dependent potent cytotoxic activity, with IC_50_ values within low nanomolar range. Other preclinical *in vitro* and *in vivo* studies have been done with plocabulin in endothelial cells (13), patient-derived colorectal cancer organoids, with dose responses very similar to ours (23), or gastrointestinal stromal tumor (GIST) patient-derived xenograft (PDX) mice (14). All of them show promising results enhancing the antitumor effect of plocabulin in different solid tumors, supporting the development of clinical trials to explore the activity of plocabulin in patients. In a first-in-human phase I clinical trial the main dose-limiting toxicity was peripheral sensory neuropathy, similarly to other tubulin-binding agents. Although an encouraging clinical benefit was observed, tolerability should be improved. Therefore, the recommended dose and schedule is not well defined yet (24).

We have not been able to establish a possible association between the *BRCA* status of the cell lines and the response to plocabulin. Only two of the cell lines tested (PEO1 and PEO16) have mutations in *BRCA2* (16), and the IC_50_ values obtained for plocabulin do not show a correlation with a *BRCA* mutated genotype. Nevertheless, future experiments with a wider series of *BRCA* mutated cell lines and the use of other drugs as PARPi could be interesting to understand this matter.

3D *in vitro* models are used in cancer research as a bridge model between *in vitro* cancer cell line cultures and *in vivo* tumor. Our data show that, when cultured as spheroids, only three cell lines remain sensitive to plocabulin in a low nanomolar range, and PEO4 is still almost three times more resistant in 3D vs. 2D culture. These results are expected, since 3D spheroids are more complex models than 2D. Their spatial architecture promotes the establishment of diffusion gradients that could modify what it is seen in monolayer, where all cells are equally exposed to drugs (25). Our group has already published a work that supports the theory that OC cells tend to be more resistant to CDDP treatment when growing on 3D (17), and similar results have also been recently described for different drugs in 3D models of colorectal cancer (26), hepatocarcinoma (27), glioblastoma (28), breast cancer (29), melanoma (30), and also in OC (25).

Cell migration plays an important role in many physiological and pathophysiological processes such as wound healing, tissue development, angiogenesis, inflammation and cancer, where the process of tumor metastasis involves invasion and migration of cancer cells (31, 32). OC predominantly metastasizes by shedding away from primary tumors and moving through the abdominal cavity in ascites fluid towards the mesothelium, where molecules such as fibronectin, laminin, type IV collagen and mesothelin promote adhesion and migration to the basement membrane/extracellular matrix (ECM) (33). Recurrent disease is very difficult to treat since it often becomes resistant to chemotherapy. Anti-migratory agents could significantly improve cancer treatment, decreasing the dependency on therapeutics and the associated side-effects by delaying the formation of metastases. Furthermore, they have been shown to sensitize migrating cells to antiapoptotic drugs (31, 34, 35).

Our results show that plocabulin can inhibit invasion of PEA1, PEA2, PEO14 and OV866(2) and migration of PEA2, PEO14 and OV866(2) HGSOC cell lines in monoculture, and in 3D spheroids of OV866(2) cells, reducing spheroid volume and cell sprouting area, even though the latter become more resistant to plocabulin in 3D. We and others have previously reported that 3D tumor spheroid-based migration assays reflect better the solid tumor microenvironment and represent both cell-cell and cell-ECM interactions. Our technique is highly reproducible and therefore appropriate for the evaluation of therapeutic drugs with anti-migratory properties (18, 31).

We and others have previously published the importance of the angiogenic process in OC and its relation to poor prognosis (36). Moreover, antiangiogenic treatment with bevacizumab is approved in OC for first line and relapse settings. Preclinical studies have reported an antiangiogenic effect of plocabulin in GIST PDX (14), and also in endothelial cells, where it inhibits the migration and invasion abilities at picomolar concentrations that suppress microtubule dynamics but do not affect cell survival (13). To our knowledge, this is the first study to evaluate the effects of plocabulin on tumor cell invasion and migration, and together with the aforementioned studies, it demonstrates an important effect in the global process of metastasis, since it can inhibit the migration of tumor and endothelial cells. All these results suggest that this secondary mechanism of action could also be beneficial in OC and should be further investigated in a combined model that includes tumor and endothelial cells.

As previously reported by Martínez-Díez et al. in a lung cancer cell line, we have observed in OV866(2) HGSOC cell line that plocabulin has a potent depolymerizing effect on microtubules, which affects cells in interphase and mitosis. In this cell line, plocabulin treatment causes the appearance of multipolar mitoses, chromosome missegregation and multinucleated cells that do not undergo anaphase/cytokinesis, forcing cells to enter senescence or apoptotic death, as seen in cell cycle experiments. Martínez-Díez et al. also reported that plocabulin-induced disorganization and fragmentation of the microtubule network could be related to the inhibition of cell migration in cells where the antiproliferative effects of this drug were not evident (11).

In this work we have also explored combinations of plocabulin with various drugs currently administered to patients with HGSOC in routine clinical practice, but none of them showed a synergistic or additive effect. Part of the reasons for the lack of interaction between the drugs is that plocabulin as a single drug is already effective at low concentrations. Combination with paclitaxel was not tested because paclitaxel and plocabulin share a similar mechanism of action (microtubule inhibitors) and dose-limiting toxicity (neurotoxicity) (24). The combinations were evaluated using SynergyFinder Plus software, a very robust and restrictive tool, since to ensure a positive synergy, all the four algorithms had to produce consistent results. To date, only one phase I clinical trial has been developed using plocabulin in combination with another drug, gemcitabine, but results are still under analysis (NCT02533674). Our results do not support the use of plocabulin in combination with other drugs, based on our combination approach. However, we do believe that it could have an interesting antitumoral activity when used in monotherapy in the treatment of OC, especially in the case of platinum-resistant relapses, where there is an unmet medical need. Our data reflect that plocabulin is effective in OC cell lines that exert different sensitivities to CDDP, but further studies are needed to confirm these findings.

As mentioned throughout the discussion, this work presents a series of strengths, such as the use of a large panel of HGSOC cell lines, the use of 3D models that better represent tumor architecture, and the analyses of drug effects in less studied processes such as migration and invasion in 2D culture and in spheroids. Moreover, we have employed a robust and restrictive tool for the exploration of drug combinations. This method did not reveal any synergies in our hands, but they cannot be discarded by complementary approaches, like animal model experiments. Nevertheless, we recognize a series of limitations. First of all, we have only been able to demonstrate migration and invasion inhibition in four cell lines in 2D and only one in 3D. Confirmation in other cell lines that migrate and invade would be desirable. Also, 3D spheroids were exposed to a maximum concentration of plocabulin of 10 nM, 100-1000 times stronger than IC_50_ values obtained in monolayer culture, but still very low, and may not be enough when scaled to *in vivo* models.

To our knowledge, this is the first work to describe the *in vitro* effects of plocabulin, a novel tubulin-binding agent, in OC. Our results show that plocabulin has potent cytotoxic activity in a panel of HGSOC cell lines, including CDDP resistance scenario, and that it inhibits migration and invasion of tumor cells and spheroids. Further clinical evaluation of this drug in OC would be warranted.

## Data Availability Statement

The raw data supporting the conclusions of this article will be made available by the authors, without undue reservation.

## Author Contributions

Concept, VH-S, AR, and MMe. Methodology, all authors. Formal analysis, VH-S, SZ, AR, and MMe. Supervision, JT, AR, and MMe. Project administration, MMe and AR. Writing and original draft preparation, VH-S, AR, and MMe. Writing, review and editing, all authors. All authors contributed to the article and approved the submitted version.

## Conflict of Interest

AG reports honoraria (Clovis, MSD, AstraZeneca, GSK, PharmaMar and Roche) and travel/accommodation/expenses (Merck Sharp and Dohme, PharmaMar, Roche, Eisai, Pfizer, Pierre-Fabre and Tesaro-A GSK Company), outside the submitted work. MM-D is employee and shareholder of PharmaMar S.A. (Madrid, Spain). AR reports honoraria and advisory/consultancy (MSD, AstraZeneca, Roche, GSK, Clovis, PharmaMar, Lilly, Amgen), research grant/funding to his institution (Eisai, PharmaMar, Roche), travel/accommodation/expenses (AstraZeneca, Tesaro: A GSK Company, PharmaMar, Roche), and speakers bureau (MSD, AstraZeneca, Roche, GSK, Clovis, PharmaMar), outside the submitted work. MMe reports honoraria (MSD, AstraZeneca and GSK), research grant/funding to her institution (Eisai and PharmaMar), travel/accommodation/expenses (AstraZeneca, GSK, PharmaMar, Roche and Pfizer), outside the submitted work.

The authors declare that this study received funding from PharmaMar S.A. (MM-D). The funder had the following involvement with the study: performed immunofluorescence experiments and reviewed draft preparation.

## Publisher’s Note

All claims expressed in this article are solely those of the authors and do not necessarily represent those of their affiliated organizations, or those of the publisher, the editors and the reviewers. Any product that may be evaluated in this article, or claim that may be made by its manufacturer, is not guaranteed or endorsed by the publisher.
