# Supplementary material for: Antitumoral Effect of Plocabulin in High Grade Serous Ovarian Carcinoma Cell Line Models
Source: Front Oncol. 2022 Mar 17;12:862321. doi: 10.3389/fonc.2022.862321 (PMC8969563; doi:10.3389/fonc.2022.862321)
Supplement: Supplementary file 1 [file Table_1.pdf]

Supplementary Table 1. Antibodies and Hoechst used for IF stains.

**Antibody / Hoechst**	**Reference**	**Dilution / Concentration**
Rabbit polyclonal anti-γ-tubulin	Sigma-T5192	1:800
Mouse monoclonal anti-α-tubulin FITC conjugated	Sigma-F2168	1:800
Alexa 594-conjugated goat anti-rabbit IgG	Thermo-A11037	1:1000
Hoechst 33258	Sigma- B2261	1 μg/ml
